# A promoter mutation in the haemagglutinin segment of influenza A virus generates an effective candidate live attenuated vaccine

**DOI:** 10.1111/irv.12274

**Published:** 2014-08-02

**Authors:** Ruth Harvey, Rachel E Johnson, Kirsty MacLellan-Gibson, James S Robertson, Othmar G Engelhardt

**Affiliations:** a National Institute for Biological Standards and Control, Medicines and Healthcare products Regulatory AgencyPotters Bar, UK; b Roche Products LimitedWelwyn Garden City, UK

**Keywords:** Candidate vaccine virus, haemagglutinin, influenza, live attenuated, promoter mutation, vaccine

## Abstract

**Background:**

Annual seasonal and pandemic influenza vaccines need to be produced in a very tight time frame. Haemagglutinin (HA) is the major immunogenic component of influenza vaccines, and there is a lot of interest in improving candidate vaccine viruses.

**Objectives:**

It has been shown elsewhere that mutations introduced in the non-coding region of influenza genome segments can upregulate protein expression. Our objective was to assess a virus based on the laboratory strain A/PR/8/34 (PR8) containing a modified 3′ non-coding region of RNA segment 4 (haemagglutinin).

**Methods:**

NIBRG-93 was generated using reverse genetics. HA protein expression and growth properties were assessed. The virus phenotype suggested that it could be a candidate for use as a live attenuated vaccine, so *in vivo* studies were performed to assess its suitability.

**Results:**

NIBRG-93 virus has enhanced haemagglutinin production and is significantly attenuated. Electron microscopy (EM) shows that the modified virus produces a large proportion of ‘virus-like particles’ that consist of budded cell membrane covered in HA but lacking M1 protein. The virus was shown to be attenuated in mice and offered complete protection against lethal challenge.

**Conclusions:**

We demonstrate that NIBRG-93 is an effective live attenuated vaccine virus protecting mice against lethal challenge and reducing virus shedding.

*Please cite this paper as:* Harvey *et al*. (2014) A promoter mutation in the haemagglutinin segment of influenza A virus generates an effective candidate live attenuated vaccine. Influenza and Other Respiratory Viruses 8(6), 605–612.

## Introduction

Influenza virus evolves rapidly due to antigenic drift or shift and annual vaccination remains the most effective protection against the disease. Because of this, the composition of influenza vaccine is reviewed and updated as necessary on a biannual basis, once for the Northern Hemisphere and once for the Southern Hemisphere. Production of seasonal vaccine can only begin once the composition has been agreed and, therefore, takes place according to a very tight schedule.^1^ Influenza vaccines are most commonly inactivated split or subunit preparations, but more recently there has been an increase in the development and use of live attenuated vaccines.^2^

Considerable research goes into improving candidate vaccine viruses (CVVs), the viruses used in the manufacture of vaccine. The characteristics required of CVVs used for inactivated vaccines versus those for live attenuated vaccines are quite different. A useful CVV for inactivated vaccine production needs to provide a high yield of stable haemagglutinin antigen, the major component of inactivated vaccine. By contrast, a useful candidate for a live attenuated vaccine has to express the recommended HA and NA antigens but retain an attenuated phenotype.

Routinely, for inactivated influenza vaccine production, high-growth reassortants are generated, traditionally by coinfection of eggs with a high-growth laboratory-adapted strain (such as PR8) and the seasonal wild-type virus of interest. Antibody selection against the PR8 HA and NA is used to select a CVV with the HA and NA of the recommended seasonal virus with high growth being selected via natural selection (and generally conferred by the ‘internal’ genes of PR8). More recently, reverse genetics has been used to generate CVVs with the required phenotypic features and has allowed research into further improvements of candidate vaccine viruses.^3^,^4^

Influenza virus is an orthomyxovirus with a segmented genome comprised of eight strands of negative-sense RNA. Each segment possesses highly conserved non-coding sequences at both the 3′ and 5′ ends. The non-coding regions are partially complimentary and form an RNA structure, described as either ‘corkscrew’ or ‘panhandle’,^5^ which is essential for the interaction of the viral polymerase with the RNA for transcription and replication. Previous studies have shown that mutation of some of the highly conserved residues in the 3′ terminal non-coding region, namely G3A, U5C and C8U, significantly increase gene expression of the mutated segment.^6^

Here, we report on a study in which we investigated the effect of introducing the G3A, U5C and C8U mutations (referred to as a 3′5′8′ mutation) into the 3′ non-coding region of the HA genome segment of PR8 to increase HA yield beyond that which can be achieved in a traditional high-growth reassortant virus. We demonstrate that the 3′5′8′ promoter mutations can be stably introduced into the HA genome segment and that, as hypothesised, the mutations lead to significantly increased HA protein expression. However, the mutations also resulted in significantly reduced titres compared with wild-type virus, abrogating its value as a CVV for inactivated vaccine. Conversely, this reduction in growth combined with high HA antigen production make this modified virus a potential candidate as a live attenuated virus vaccine. When this virus was tested in a mouse model, it was highly attenuated compared with A/PR/8/34 and more significantly provided complete protection against challenge by wild-type virus.

## Materials and methods

### Modified HA plasmid and virus

Mutation of the 3rd, 5th and 8th nucleotide (referred to as a 3′5′8′ mutation) of the 3′ non-coding region of the HA-encoding genome segment of PR8 was achieved using standard site-directed mutagenesis procedures. The modified HA gene was inserted into the reverse genetics pPST vector using unique SapI restriction sites using standard cloning protocols. The resulting rescued virus, NIBRG-93, was derived using reverse genetics^3^ and comprises the 3′5′8′ modified HA segment with all other segments deriving from wild-type PR8.

### Confirmation of 3′5′8′ sequence by RNA ligation

To confirm the presence of the 3′5′8′ mutation, the entire HA segment of NIBRG-93 HA was sequenced. Sequencing of the 5′ and 3′ non-coding regions was achieved after circularisation of NIBRG-93 viral RNA, performed as previously described.^7^

### Virus concentrates

Viruses were grown in 11-day-old embryonated hens' eggs and purified as previously described.^4^

### Deglycosylation using PNGase F and SDS-PAGE analysis

Deglycosylation was achieved using PNGase F (New England Biolabs) prior to samples being analysed by SDS-PAGE analysis as previously described.^8^

### BCA assays

Total protein content of virus concentrates was determined using a BCA assay kit (Pierce) according to manufacturer's instructions. The microtitre plate protocol was followed using BSA standards (provided with the kit).

### Plaque assays

Viruses were diluted to 10^−4^, 10^−5^ and 10^−6^ in PBS and 200 μl of each dilution was used to infect wells of a six-well plate seeded with MDCK cells. Plates were incubated at room temperature for 40 minutes to allow virus absorption. Plates were overlaid with a media/Avicel suspension (1× MEM, 0·2% BA, 1× L-glutamine, 0·01% Dextran, 0·0001% TPCK Trypsin, 0·18% sodium bicarbonate, 1·2% Avicel type RC-581) and incubated for 72 hours at 35°C with 5% CO_2_ after which time the overlay was removed and cells were fixed with 3% formaldehyde solution. Cells were then stained with naphthalene black. In assessing temperature sensitivity, plaque assays were additionally performed at 33°C and 38°C.

### Mice

Female BALB/c mice (adult) were purchased from Charles River Laboratories (Kent, UK) and housed according to UK regulations. The study was approved and conducted according to the Animal (Scientific Procedures) Act 1986. All procedures were carried out according to UK Home Office Licence regulations, and the study was approved by NIBSC Ethical Review Process.

The study comprised two parts: firstly, assessment of attenuation and secondly, protective efficacy after challenge. A group of 15 mice was inoculated intranasally (IN) with 10^5·5^ TCID_50_ wild-type A/PR/8/34; three groups of 35 mice were inoculated IN with 10^4·5^, 10^5·5^ or 10^6·5^ TCID_50_ NIBRG-93. Two groups of 15 mice were used as control groups and were inoculated intramuscularly (IM) with 15ugHA/mouse inactivated PR8 or PBS only. After inoculation, and throughout the entire course of the study, mice were weighed and observed daily for clinical signs of disease (ruffled fur, neurological signs and respiratory symptoms). Mice were euthanized following >20% weight loss or poor general condition. At days 2, 4, 10 and 30 post-inoculation, 5 mice per group from IN-inoculated animals were euthanized for virus recovery. For the second part of the study, at day 28 post-inoculation, remaining mice were challenged IN with 10^5·5^ TCID_50_ PR8. At days 30 and 32 post-inoculation, nasal washes were performed to monitor viral shedding.

### Virus recovery from samples

The presence of virus in nasal washes, nasal turbinates and lungs of mice was quantified by TCID_50_ in MDCK cells as previously described.^9^ Briefly, tissue samples were homogenised, and dilutions of samples were incubated on confluent monolayers of MDCK cells at 35°C for 72 hours (5% CO_2_). A haemagglutination assay with 0·7% turkey red blood cells was used to detect virus in the supernatant. Wells were scored for the presence or absence of virus, and the titre expressed as TCID_50_ per ml for nasal washes, or per gram of tissue. Titres were calculated by the Spearman-Karber method.^10^,^11^

### Haemagglutination inhibition

Sera from days 1 (pre-bleed), 18 (post-immunisation) and 42 (post-challenge) were tested in HI assay by standard methods using 0·7% turkey red blood cells. The serum HI titre against PR8 was expressed as the reciprocal of the highest dilution at which haemagglutination was inhibited.

### Electron microscopy

Samples were diluted 1:50 (PR8) and 1:10 (NIBRG-93) in PBS and were applied to a freshly glow discharged Quantifoil grid (Agar scientific, Stansted, Essex, UK). Grids were blotted on both sides for 3·8s and were plunged into liquid ethane held at −172°C in a Gatan CP3 (Gatan Inc., Pleasanton, CA, USA). Grids were transferred at cryogenic temperatures into a 914 holder (Gatan) cooled to −175°C; images were acquired on a JEM2100 [JEOL (UK) Ltd., Welwyn Garden City, Hertfordshire, UK] under low-dose conditions using a US4000 camera running digital micrograph software (Gatan).

### Statistical analysis

Statistical analysis was performed using minitab 16 statistical software (Minitab Inc., State College, PA, USA). The analysis (anova) was performed using a general linear model with the Tukey method for pair-wise comparisons.^12^

## Results

G3A, U5C and C8U (3′5′8′) promoter mutations of the 3′ non-coding region are known to upregulate gene expression of the segment in which they occur.^6^ In the current study, the 3′5′8′ mutation was introduced into the 3′ non-coding region of the HA genome segment of PR8, which was successfully rescued into a viable virus, named NIBRG-93, using reverse genetics. To assess the stability of the mutation, the virus was passaged 10 times in embryonated hens' eggs, and the sequence of the HA of the final passage (VE12) was analysed. The passaged virus was confirmed to contain the modified 3′5′8′ sequence in the 3′ non-coding region of segment 4. A single point mutation of G to T at nucleotide 14 was found at VE12, but this was not present in the VE3 virus used for all the studies. The point mutation did not alter the attenuated phenotype of the virus as measured by HA titre of the virus at each passage (data not shown).

**Figure 1:**
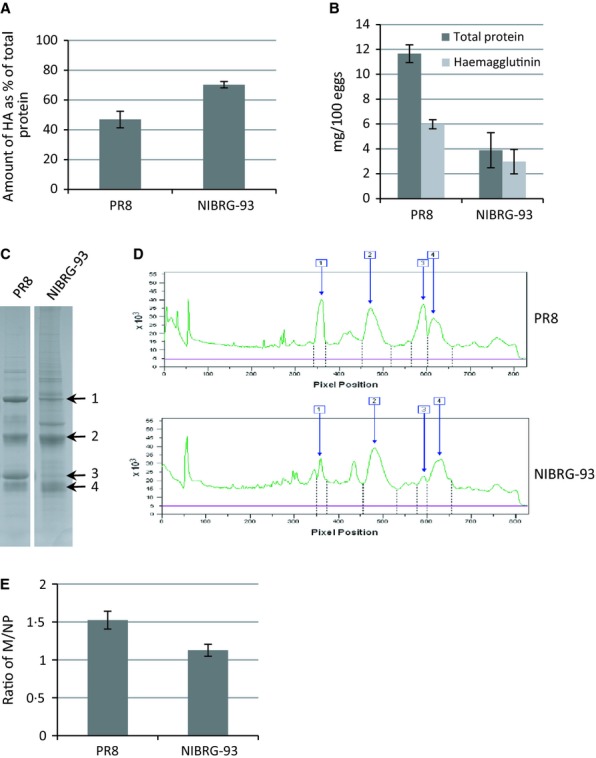
Yield analysis of NIBRG-93. (A) Quantitation of haemagglutinin (HA) content. Quantitation was carried out using image quant software. For each sample, the HA content was calculated as described in Materials and methods. (B) Total protein yield (Dark grey bars). The total protein yield for each virus was measured using a BCA assay as described in Materials and methods. HA protein yield (light grey bars). The yield of HA (mg HA/100 eggs) was calculated based on total protein and the %HA (as shown in panel A). For all data in A and B, each virus was tested on at least 3 occasions and error bars show standard deviations. (C) SDS-PAGE analysis of virus concentrates. Samples were reduced and deglycosylated; major virus proteins are identified: 1, NP; 2, HA1; 3, M1; and 4, HA2. (D) Scan of gel lanes using image quant software. Viral proteins are indicated by numbers as above. (E) Ratio of M1 to NP. Quantitation was carried out using image quant software.

The HA content of NIBRG-93 and PR8 was assessed as previously described.^8^ The results (Figure 1A) showed a dramatic and statistically significant increase in the HA content of NIBRG-93, with HA amounting to 70% of viral protein compared with 45–50% for the PR8 wild-type virus. The total viral protein content of purified virus concentrates is also an important contributor to the yield of HA antigen during vaccine production.^13^,^14^. For PR8 and NIBRG-93, (Figure 1B dark grey bars) the total protein yield was 11·6 mg/100 eggs and 3·9 mg/100 eggs, respectively (*P* < 0·05). Combining total protein yield and HA content data (Figure 1B light grey bars), the calculated yield of HA per 100 eggs was 6 mg for PR8 and 3 mg for NIBRG-93 (*P* < 0·05). Figure 1C shows SDS-PAGE analysis of de-glycosylated PR8 and NIBRG-93, while Figure 1D shows the quantitative analysis trace through these gel lanes. The relative increase in the HA content as compared to the NP and M1 bands is clearly discernible. Also, the analysis suggests a significant reduction in the M1 protein content for NIBRG-93 as compared to PR8. To investigate whether the increase in the HA content led to changes in the other major viral proteins, the NP and M1, we calculated the ratio of M1/NP for PR8 and NIBRG-93. The results (Figure 1E) showed a small but significant reduction in the M1/NP ratio for NIBRG-93 as compared to PR8, from 1·53 for PR8 to 1·21 for NIBRG-93 (*P* < 0·05), indicating a statistically significant reduction in the M1 protein in NIBRG-93 virus particles.

**Figure 2:**
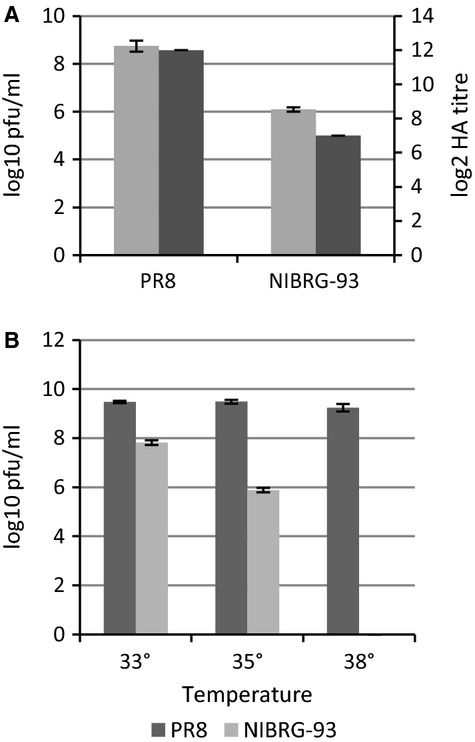
Growth characteristics of PR8 and NIBRG-93. (A) Titre of viruses in eggs at 35°C. Pale grey bars show pfu/ml and dark grey bars show haemagglutinin titre. (B) Titre of PR8 (dark grey) and NIBRG-93 (light grey) in eggs at various temperatures. Each data point is the average of at least three repeats and error bars show standard deviation.

The titres of NIBRG-93 and PR8 grown in eggs were assessed by plaque and HA assays of allantoic fluid and show that the infectious titre of NIBRG-93 was significantly (*P* < 0·05) lower than that of wild-type PR8 with virus titres of 10^6^ pfu/ml versus 10^9^ pfu/ml, respectively (Figure 2A). HA titres showed a similar result, with NIBRG-93 growing to an HA titre of 128 and PR8 growing to a titre of ≥4096. Further comparison of the two viruses was carried out by performing plaque assays at different temperatures (33°C, 35°C and 38°C). PR8 grew to a similar titre, of 10^9^ pfu/ml, at all temperatures, while NIBRG-93 replication displayed temperature sensitivity, with titres of 10^8^ pfu/ml at 33°C, 10^6^ pfu/ml at 35°C and no detectable plaques at 38°C (Figure 2B).

**Figure 3:**
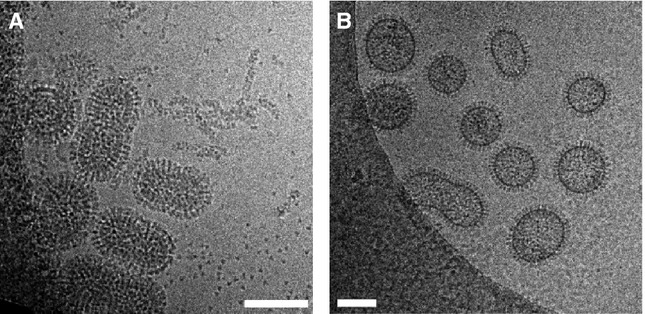
Morphology of virus particles. Virus particles were visualised using cryo-electron microscopy. Representative electron micrographs of wild-type PR8 (A) and NIBRG-93 (B) are shown. Scale bar (in white) denotes 100 nm.

Electron microscopy (EM) analysis of PR8 (Figure 3A) and NIBRG-93 (Figure 3B) showed distinct morphologies for the two viruses. PR8 had typical pleomorphic particles of approximately 100 nm diameter; in contrast, NIBRG-93 was considerably less pleomorphic being comprised primarily of spherical particles approximately 100 nm in diameter. HA protein spikes are visible in PR8 particles, studded into the lipid envelope of the virus. The lipid envelope can be seen as a thick ‘fuzzy’ layer due to the M1 protein associated with the membrane^15^ and lining the inside of the virus particles. In contrast, the membrane of NIBRG-93 particles was atypical, appearing thinner, denser and sharper compared with PR8, suggestive of a lipid bilayer lacking M1 (matrix protein).

**Figure 4:**
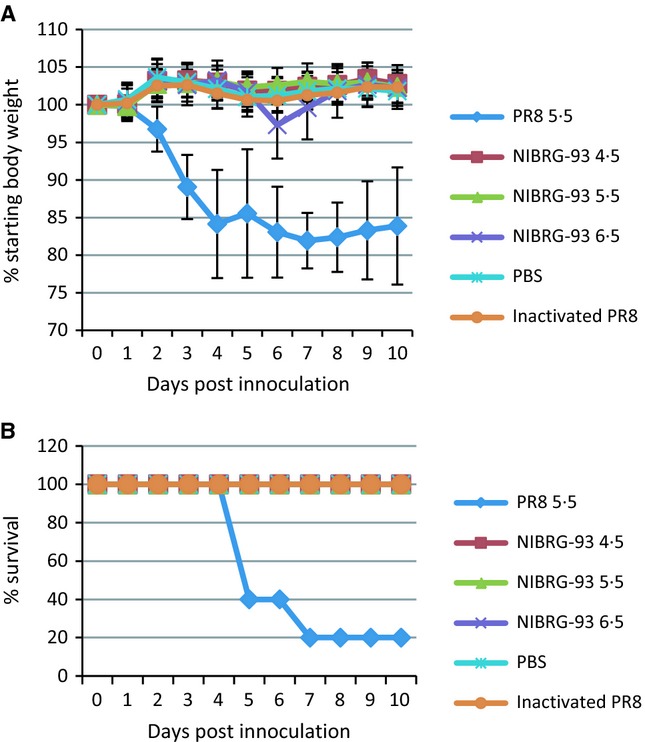
Attenuation of NIBRG-93 *in vivo*. (A) Weight loss and (B) survival following infection with PR8 or NIBRG-93. 15 mice were inoculated IN with 10^5·5^ TCID_50_ wild-type A/PR/8/34, three groups of 35 mice were inoculated IN with 10^4·5^, 10^5·5^ or 10^6·5^ TCID_50_ NIBRG-93. Two groups of 15 mice were used as control groups and were inoculated IM with 15ugHA/mouse inactivated PR8 or PBS only. After inoculation, and throughout the entire course of the study, mice were weighed and observed daily for clinical signs of disease. Error bars show standard deviations.

**Figure 5:**
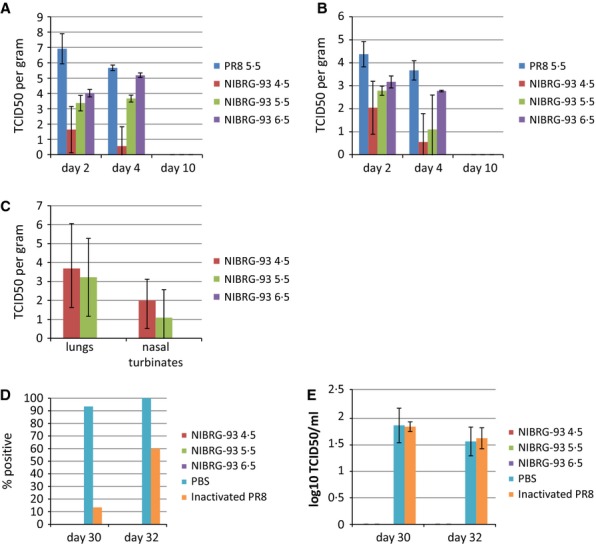
Titration of virus recovered from tissues and nasal washes. Virus recovery was assessed using TCID_50_ titration of samples. (A) Virus recovery from lungs. (B) Virus recovery from nasal turbinates. (C) Virus recovery from lungs and nasal turbinates day 30 post-inoculation (2 days post-challenge). (D) Percentage of animals with virus recovered from nasal washes. (E) TCID_50_ of virus recovered from positive nasal washes.

With NIBRG-93 showing increased HA content but low overall HA yield/100 eggs, as well as decreased infectivity, its potential as a live attenuated vaccine was determined in mice. To assess attenuation, one group of 15 mice was inoculated intranasally (IN) with 10^5·5^ TCID_50_ *wt* PR8 (group A), three groups of 35 mice each were inoculated IN with 10^4·5^ TCID_50_ (group B), 10^5·5^ TCID_50_ (group C) and 10^6·5^ TCID_50_ of NIBRG-93 (group D). Two groups of 15 mice each were used as control groups and were inoculated intramuscularly (IM) with PBS (group E) or 15ugHA/mouse inactivated PR8 (group F). In mice infected with *wt* PR8 (group A), 80% of the mice lost 20% of their body weight by day 4 post-inoculation (Figure 4A). There was a transient minor weight loss (<5%) at day 6 post-inoculation in group D (infected with the highest level of NIBRG-93). All other groups showed no weight loss. Mice infected with *wt* PR8 also showed significant signs of disease by day 4 with only 20% survival (Figure 4B). All other groups showed 100% survival with no signs of disease. Mice from groups A–D were sacrificed on days 2, 4 and 10 (5 per time point) for virus recovery from lungs (Figure 5A) and nasal turbinates (Figure 5B). The highest titres of virus (10^6·9^ TCID_50_ per gram of lung and 10^4·4^ TCID_50_ per gram of nasal turbinate) were found in animals infected with PR8, while significantly less (*P* < 0·05) virus was found in lungs and nasal turbinates of animals infected with NIBRG-93 (10^4·3^ TCID_50_ per gram and 10^3·1^ TCID_50_ per gram respectively for highest dose of NIBRG-93). There was a correlation between the dose of NIBRG-93 given and virus recovery from tissues. On day 10, no virus was recovered from any of the animals. Approximately 80% of the PR8-infected animals showed severe clinical signs and were humanely euthanized before day 10; all remaining animals cleared the infection.

**Figure 6:**
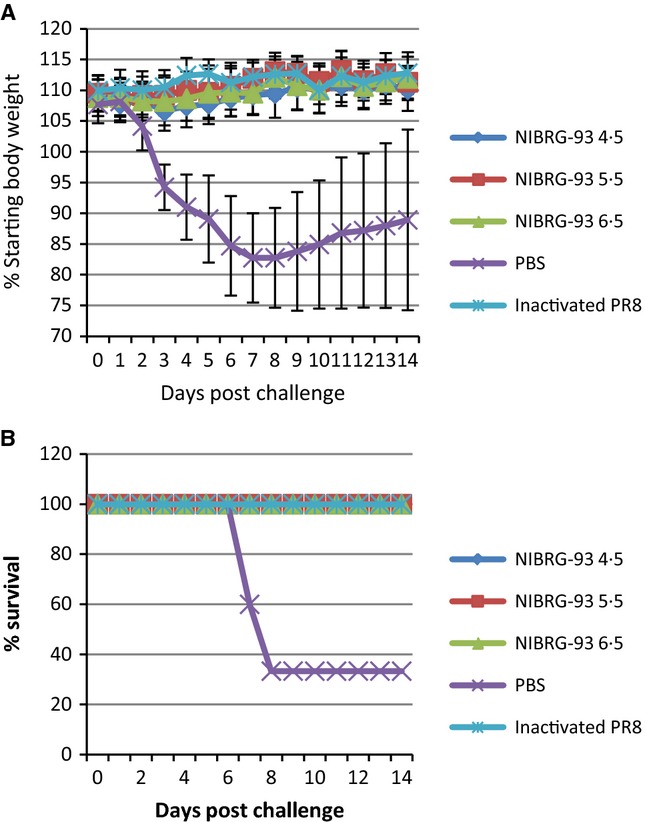
Protection from viral challenge. (A) Weight loss and (B) survival following challenge with *wt* PR8. At day 28 post-inoculation, mice were challenged with a lethal dose of 10^5·5^ TCID_50_ *wt* PR8 virus. Mice were weighed and observed for clinical signs of disease. Error bars show standard deviations.

Following the assessment of attenuation, all remaining animals in groups B, C, D, E and F were challenged with *wt* PR8 at day 28 post-inoculation, using the same dose as used initially for group A. Five mice from groups B, C and D were sacrificed at day 30 of the study (2 days post-challenge) to assess viral load. Nasal washes were performed on all mice from all groups on days 30 and 32 (2 and 4 days post-challenge, respectively) to assess viral shedding. Virus was recovered from lungs and nasal turbinates (Figure 5C) from groups B (inoculated with 10^4·5^ TCID_50_ NIBRG-93) and C (inoculated with 10^5·5^ TCID_50_ NIBRG-93). No virus was recovered from the lungs and nasal turbinates of group D. The body weight and survival data (Figure 6) show that 100% of mice from all groups except the PBS only control (group E) were protected from disease and death following challenge with *wt* PR8. There was no weight loss or other sign of illness in protected mice. The results of the nasal washes taken at day 30 (day 2 post-challenge) showed that virus was being shed from 90% of mice in control group E and 12% of mice in group F (Figure 5D); by day 32 (day 4 post-challenge), this had risen to 100% and 60%, respectively, while no virus was detected in nasal washes from any of the other groups. The virus in the positive nasal washes was titrated by TCID_50_ (Figure 5E), and while there was a significant difference between the number of mice shedding virus, the titres of virus shed from the two groups were not significantly different, remaining between 1·5 and 2 TCID_50_/ml from groups E and F on both days 30 and 32.

**Figure 7:**
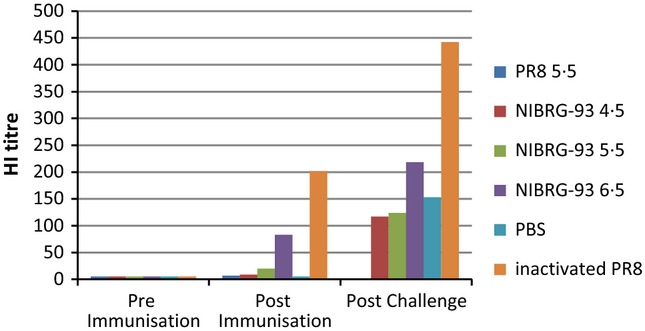
Haemagglutination inhibition antibody titres from blood samples pre-immunisation, post-immunisation and post-challenge. Data shown are geometric mean titres of antibody responses from all animals within a group.

At days 18 (pre challenge) and 42, post-inoculation (14 days post-challenge) blood samples from all surviving mice were tested for antibody response by haemagglutination inhibition assay. At day 18, mice from groups A (10^5·5^ TCID_50_ PR8) and B (10^4·5^ TCID_50_ NIBRG-93) showed no antibody response (Figure 7), whereas groups C and D (10^5·5^ and 10^6·5^ TCID_50_ NIBRG-93 respectively) had an antibody response, with group D titres being higher than those in group C. Group E (PBS only) showed no antibody response and group F (IM-inactivated PR8) produced high titres of anti-PR8 antibody.

After challenge, all groups (note: group A was not included in the challenge experiment) showed an antibody response or an increase in antibody titre, with the highest dose of NIBRG-93 (group D) showing a higher response than groups B, C or E. The highest response was seen in the control group F, which had received IM vaccination with inactivated PR8.

## Discussion

Vaccination is the most effective way of mitigating influenza, and the annual cycle of seasonal vaccine production after strain selection occurs under pressure due to the short time frame. In the event of a pandemic there would be huge global demand for vaccine in the shortest time frame, therefore, development of new approaches to increase the number of vaccine doses remains an important area of research. To this end, we investigated the effect of adding a mutation previously shown to upregulate gene expression^6^ to the 3′ non-coding region of segment 4 which encodes HA. The aim of this was to increase the amount of HA produced by the resulting virus and so to create a candidate vaccine virus that more efficiently produced the HA antigen, the major component of influenza vaccines. Having successfully rescued a viable virus containing the desired mutation (NIBRG-93) and shown that through repeated passage in eggs the mutation was stable, we demonstrated that, as hypothesised, this new virus produced more HA when measured as a percentage of total virus protein, with an increase from 45% to 70% HA content, as compared to *wt* PR8 (Figure 1A). However, NIBRG-93 demonstrated a significant reduction in growth, achieving titres of three logs less than PR8 which abrogated the benefit of a considerably increased HA content (Figure 2A). The growth of NIBRG-93 was also shown to be temperature sensitive (Figure 2B), with reduced growth at 35°C compared with 33°C and no growth at all at 38°C. In contrast, PR8 growth was equivalent at 33, 35 and 38°C. The dramatic reduction of virus growth due to a mutation in the 3′ non-coding region of only one segment of the virus has also recently been observed by Belicha-Villanueva *et al*.^16^ who published a study in which they introduced a similar ‘super-promoter’ mutation into either segment 2 (PB1) or segment 3 (PA). They demonstrated that this led to enhanced protein expression from the modified segment, but that replication of the viruses containing the mutations was attenuated; the authors commented that this highlighted the need for a balance between viral replication, protein expression and assembly.

Further investigation into the phenotype of NIBRG-93 by EM suggested that it produced a large number of atypical virus-like particles (VLPs) consisting of cell membrane covered in HA antigen but with little or no matrix protein lining the inside of the membrane (Figure 3). The presence of such VLPs would explain the reduction in the amount of M1 protein relative to other viral protein measured by SDS-PAGE (Figure 1C–E). NIBRG-93 also expresses NA, and it has been shown previously that HA protein can bud to form VLPs in the absence of M1 protein as long as neuraminidase activity is present.^17^

The attenuation in growth of NIBRG-93 led us to hypothesise that it could be a good candidate as a live attenuated vaccine virus rather than a vaccine virus for inactivated vaccine production. NIBRG-93 was highly attenuated in mice causing no illness or weight loss even at an infectious dose, ten times a dose of PR8 that causes 80% mortality (Figure 4). Furthermore, prior intranasal inoculation with NIBRG-93 protected animals against subsequent lethal challenge with PR8 (Figure 6). Also, after challenge, no virus could be recovered from lungs or nasal turbinates of mice previously inoculated with the highest dose of NIBRG-93. Thus, inoculation of 10^6·5^ TCID_50_ NIBRG-93 was sufficient to protect fully against PR8. It is interesting to note also that mice given the live attenuated NIBRG-93 showed no shedding of virus after challenge, whereas mice given the IM-inactivated PR8 shed virus for a number of days after challenge. Thus, while the control group of mice vaccinated with inactivated PR8 intramuscularly had a significantly higher HI antibody response than that observed in mice infected with live NIBRG-93, and protection from disease and shedding was equal or better for NIBRG-93 as compared to vaccination with inactivated virus. It is widely acknowledged that HI titres are not a correlate of protection for live attenuated influenza vaccines.^18^ It has been shown in human clinical trials that live attenuated influenza vaccines provide protective immunity against challenge irrespective of the levels of induced serum antibodies, sometimes inducing no measureable rise in serum antibodies at all.^18^

Overall these data demonstrate the potential of a new prototype live attenuated vaccine virus. A similar study was recently published introducing the ‘super-promoter’ mutation to segment 4 (HA) of an already attenuated influenza virus.^19^ In that study the authors also demonstrated the suitability of the modified virus as a candidate live attenuated vaccine virus and showed effective protection in mice. The difference in our study is that the promoter mutation in the 3′ non-coding region of segment 4 alone was sufficient to lead to the attenuated phenotype. It would be interesting to apply the same approach to other viruses, either wild-type viruses with the segment 4 promoter mutation or 6:2 reasssortants with six backbone segments of PR8 and the NA and 3′5′8′ modified HA from more modern seasonal strains.

The finding that modification of the 3′ non-coding region of the HA segment led to a virus that produces a large number of atypical virus-like particles was a surprising and interesting discovery. We do not understand the mechanism underlying this observation, but speculate that overexpression of HA leads to aberrant budding in infected cells. It would be interesting to define the mechanism behind this phenotype and to examine the phenomenon in other influenza viruses.
